# Prognostic and Predictive Biomarkers in Patients with Locally Advanced Rectal Cancer (LARC) Treated with Preoperative Chemoradiotherapy

**DOI:** 10.3390/jcm11206091

**Published:** 2022-10-16

**Authors:** Alfonso Martín-Carnicero, Enrique Ramalle-Gomara, Susana Rubio-Mediavilla, Martina Alonso-Lago, Miriam Zorrilla-Larraga, Isabel Manrique-Abós, María E. de las Heras-Dueña, Ignacio M. Larrayoz, Alfredo Martínez

**Affiliations:** 1 Medical Oncology Department, Hospital San Pedro, 26006 Logroño, Spain; 2 Department of Epidemiology, La Rioja Government, 26071 Logroño, Spain; 3 Pathology Service, Hospital San Pedro, 26006 Logroño, Spain; 4 Healthcare Center, 26143 Murillo de Río Leza, Spain; 5 Biomarkers and Molecular Signaling Group, Center for Biomedical Research of La Rioja (CIBIR), 26006 Logroño, Spain; 6 Unidad Predepartamental de Enfermería, Universidad de La Rioja (UR), 26006 Logroño, Spain; 7 Angiogenesis Group, Center for Biomedical Research of La Rioja (CIBIR), 26006 Logroño, Spain

**Keywords:** locally advanced rectal cancer, neoadjuvant chemoradiotherapy, prognostic biomarkers, predictive biomarkers, hemoglobin, lymphocyte/monocyte ratio, platelet/lymphocyte ratio, positive nodes after surgery, KRAS mutations, bilirubin

## Abstract

Neoadjuvant chemoradiotherapy (CRT) is one of the standards of care in locally advanced rectal cancer (LARC). This retrospective study examines clinical, analytical, and pathological parameters collected from 77 patients with locally advanced (cT3-4 or cN+) rectal carcinoma diagnosed between 2007 and 2017 at our institution that were treated with preoperative CRT and surgery. In the prognosis analysis, lower hemoglobin levels (*p* = 0.008), lower lymphocyte/monocyte ratio (LMR) (*p* = 0.011), and higher platelet/lymphocyte ratio (PLR) (*p* = 0.029) in the second determination (Hb2, LMR2 and PLR2) were associated with the relapse group. The number of positive nodes after surgery (N+) showed a statistically significant association with relapse (*p* = 0.012). KRAS mutations were associated with a worse prognosis for 5 years progression-free and overall survival (*p* = 0.005 and 0.022; respectively). We propose a prognostic model based on four parameters (number of positive lymph nodes after surgery, hemoglobin levels, LMR, and PLR after neoadjuvant therapy) that can be a useful tool to estimate relapse risk. Moreover, bilirubin could be a useful parameter to predict the response to neoadjuvant CRT.

## 1. Introduction

Worldwide, colorectal cancer (CRC) is the third malignancy in incidence among men, after prostate and lung cancer, and the second in women after breast cancer, with more than 1.9 million new cases per year and an estimated mortality of 935,000 deaths in the year 2020 [1]. Approximately 30% of CRCs are located in the rectum. Currently, one of the standard treatments for locally advanced rectal cancer (LARC) is neoadjuvant chemoradiotherapy (nCRT) followed by surgery based on total mesorectal excision [2,3,4]. This approach has been shown to reduce local recurrence rates and toxicity compared to the administration of adjuvant CRT, although no differences in overall survival (OS) have been shown.

Around 60% of patients treated with nCRT will achieve a certain degree of pathological response, with a variable percentage of pathological complete responses (pCR) ranging from 10–20% [5,6]. This tumor regression grade has been considered a surrogate marker of survival regardless of clinicopathological parameters [7,8]; however, the high number of response grading scores [9,10,11,12,13,14] discourages the use of this parameter in daily clinical practice. In addition, and except for clinical-pathological parameters, there are currently no validated biomarkers that allow us to predict either the response to combined nCRT treatment or to determine the risk of relapse in our patients. Numerous authors have tried to find molecular biomarkers either in blood or tissue based on inflammation parameters, DNA or miRNA microarrays, mutations and DNA methylation patterns, circulating tumor cells, and even metabolites, all of them with sometimes contradictory results and never validated in large prospective series [15,16,17]. In this regard, the tumor heterogeneity characteristic of rectal cancer could play an important role in this discrepancy.

The aim of this study was to correlate clinical, laboratory, and pathological data with response to treatment and survival in a cohort of patients with LARC treated at our center with nCRT followed by surgery, in order to identify prognostic factors for survival and predictive factors for response to CRT.

## 2. Materials and Methods

### 2.1. Patients

Initially, 92 patients with LARC (T3/4 and/or N+) treated with nCRT between March 2007 and August 2017 were pre-selected, but 15 patients had to be excluded since their histopathological samples were deteriorated. As a result, a total of 77 patients were included and analyzed. The study was approved by the Medical Research Ethics Committee of La Rioja (CEICLAR, protocol number 129) and all patients signed the informed consent before inclusion.

### 2.2. Treatment Protocol

All patients were treated with a long course of radiotherapy (44–45 Gy) and concomitant chemotherapy for five weeks. Continuous infusion of 5-fluorouracil (5FUci: 225 mg/m^2^/day), capecitabine (875 mg/m^2^/12 h every day) and FOLFOX-6 (5FU bolus 400 mg/m^2^), leucovorin (400 mg/m^2^), oxaliplatin (85 mg/m^2^), and 5FUci (2400 mg/m^2^ every two weeks) were the applied chemotherapy schemes. Surgery was performed according to the principles of total mesorectal excision 7–9 weeks after completion of chemoradiotherapy. After surgery, all patients were offered adjuvant chemotherapy with 5FU (continuous infusion or capecitabine), either as monotherapy or associated with oxaliplatin, depending on the pathological stage.

### 2.3. Evaluation

**Figure 1:**
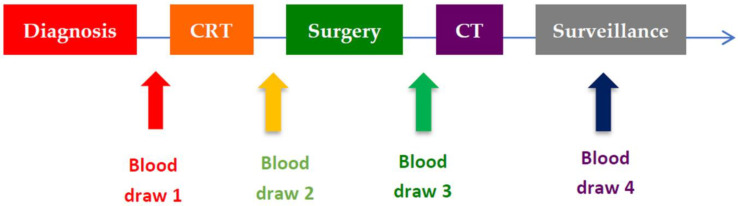
Timeline of blood draws. CRT: chemoradiotherapy; CT: chemotherapy.

Clinical data collected included age, sex, distance from the anal margin, smoking history, and clinical stage according to the 8th edition of the AJCC (cTNM) as confirmed by body CT and pelvic MRI. We calculated the days from the diagnosis to the start of CRT (CRTDD), the days from the end of CRT to surgery (CRTSD), the days from diagnosis to surgery (DSD), and the days from surgery to the beginning of adjuvant CRT (aCRT), if received (SCTD). In addition, progression-free survival (PFS) was calculated, defined as the time interval between the start of CRT and the date of relapse, and OS as the time elapsed from the start of CRT to the last date of study closure. All patients included in the study had four blood tests: the first or baseline before the start of CRT, the second prior to surgery, the third after surgery and prior to adjuvant chemotherapy treatment if the patient received it. The fourth was after the end of adjuvant CT or, otherwise, at the first review (Figure 1).

From each blood draw, the counts of leukocytes, neutrophils, monocytes, hemoglobin, and platelets were recorded. In addition, the neutrophil/lymphocyte ratio (NLR), the lymphocyte/monocyte ratio (LMR), and the platelet/lymphocyte ratio (PLR) were calculated. From the basal analysis, the values of glucose, urea, creatinine, uric acid, sodium, potassium, triglycerides, cholesterol, LDH, GOT, GPT, bilirubin, alkaline phosphatase, ferritin, iron, calcium, total proteins, albumin, and CEA (carcinoembryonic antigen) before CRT were also obtained.

KRAS mutations and microsatellite instability (MSI) were determined from fresh frozen and formalin-fixed paraffin-embedded tumor lesions. To evaluate the degree of tumor regression (TRG) to CRT, the modified Ryan classification system recommended by the American College of Pathologists [13] was used. Tumors were classified according to the 8th edition of the AJCC-TNM classification [12] after neoadjuvant treatment (ypTNM). Data on other known prognostic factors such as vascular, lymphatic, and perineural permeation, as well as the number of affected and resected lymph nodes, were also recorded.

### 2.4. Statistical Analysis

The characteristics of the patients were described by means and standard deviations, medians and interquartile ranges, or frequencies and percentages, depending on the normality and nature of the variables.

Patients were classified according to recurrence and tumor regression grade to make comparisons between groups using comparison tests for independent samples such as Student’s t, Mann–Whitney U, or Fisher tests. The logistic regression model was also used to study the influence of the different variables on relapse and response, and for which ROC curves were used to define a predictive and prognostic model. The Kaplan–Meier method was used for the analysis of OS and progression-free survival (PFS) and the log-rank test and the Cox proportional hazards model were used to compare the survival between groups. Differences were considered statistically significant for a two-tailed test when *p* < 0.05. Version 20.0 of SPSS Inc., Chicago, IL, USA was used for all statistical analyses.

## 3. Results

### 3.1. Patients Characteristics

The median (Q1–Q3) follow-up was 10.7 (3.72–13.67) years. The study sample consisted on 77 patients, 21 (27.3%) women and 56 (72.7%) men. The median age was 62 (30–79) years. A total of 73 patients (94.8%) had a clinical stage ≥T3N0. The median distance from the tumor to the anal margin was 7 (1–15) cm; the majority were in the middle and lower rectum (88.3%). A total of 41 (53.2%) patients had a history of smoking (active smokers or former smokers). The median preoperative CEA was 5.4 (0.6–77.5) ng/mL. The median number of days from diagnosis to the start of CRT was 55 (14–100) days, while the days from CRT to surgery, from diagnosis to surgery, and from surgery to start of aCRT was 49, 141, and 45 days, respectively. A total of 52 (67.5%) patients were treated with 5FUic, 24 (31.2%) patients received capecitabine, while 1 of the patients received FOLFOX-6 (1.3%), all of them concomitant with radiotherapy. A total of 33 (42.9%) patients underwent abdominoperineal amputation at surgery. A total of 36 (46.8%) patients presented ypTNM ≥ T3N0 (Table 1).

Histopathologically, 9 (12%) patients had microsatellite instability (MSI) and 19 (25%) patients had KRAS mutations. Regarding the tumoral response grading, we have data from 74 of the 77 (96.1%) patients. Of these, 28 (36.4%) patients presented TRG0-1 (7 TRG0), while 46 (59.7%) presented TRG2-3 (13 TRG3). After surgery, analysis of the surgical specimen showed that 8 (11.4%) patients had vascular permeation, 9 (11.7%) lymphatic permeation, and 15 (19.5%) perineural permeation. A total of 20 (26.7%) patients had lymph node involvement, with an average of 9.28 (0–32) isolated nodes and a mean of 1.39 (0–29) infiltrated nodes (Table 1).

### 3.2. Prognosis Factors

Throughout the follow-up period, 32 (41.6%) patients relapsed, while 45 (58.4%) did not. In the univariate analysis of clinical and pathological characteristics (Table 1), statistically significant differences were only found for KRAS mutations (*p* = 0.030), extramural venous invasion (EMVI) (*p* = 0.048), lymphatic invasion (*p* = 0.022) and perineural invasion (*p* < 0.001), lymph node involvement (*p* < 0.001), and the median number of infiltrated lymph nodes (*p* = 0.001). Regarding the pathologic response criteria to chemoradiation therapy, there were no differences in tumor regression score (TRG), although statistically significant differences were observed in the pathological TNM staging (ypTNM ≥ T3N0; *p* = 0.020). 

In the univariate analysis of hematological data (Table 2 and Table 3), statistically significant differences were obtained in hemoglobin levels at the second (Hb2) and third (Hb3) blood draws (*p* = 0.008 and 0.007, respectively), in the lymphocyte/monocyte ratio at the second blood draw (LMR2) (*p* = 0.011), in the platelet/lymphocyte ratio also at the second blood draw (PLR2) (*p* = 0.029), and in the ferritin levels (*p* = 0.025) in the biochemical data (Supplementary Table S1).

**Figure 2:**
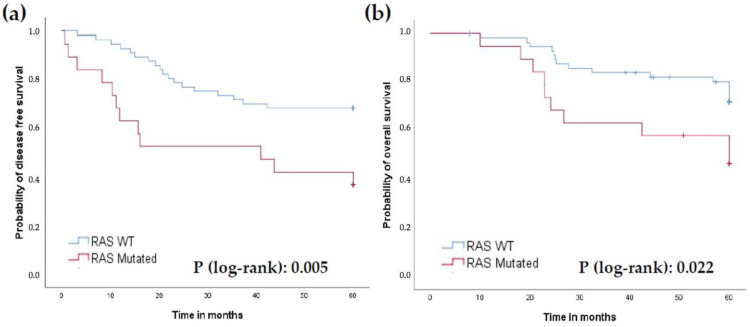
Prognostic role of KRAS status. (**a**) Progression-free survival (PFS). (**b**) Overall survival (OS).

Kaplan–Meier survival analyses demonstrated that patients with mutant KRAS had reduced 5-year PFS (36.8% vs. 68.4%) and OS (47.4% vs. 73.7%) compared to those with wild type (WT) KRAS (*p* = 0.005 and 0.022, respectively) (Figure 2a,b). 

Given these findings, and in order to find a model that could be used in clinical practice, we decided to find the optimal cut-off point for Hb2, PRL2, and LMR2, since all of them could be determined simultaneously at the second blood draw. From here, we calculated the Youden index [18] from the ROC curves of each variable, obtaining cut-off points of 12.3 g/dL for Hb2, 1.26 for LMR2, and 229.50 for PLR2.

**Figure 3:**
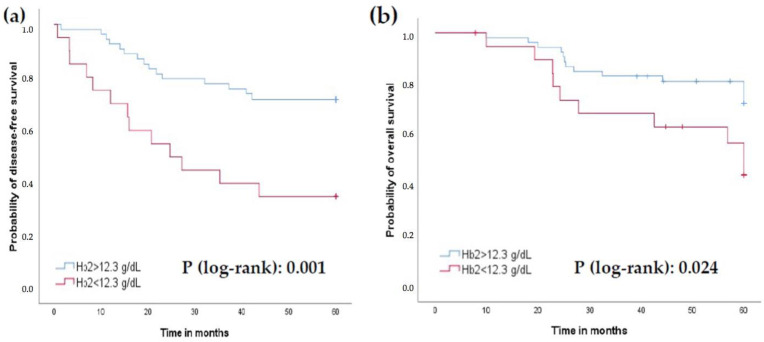
Prognostic role of Hb2 levels with a cut-off value of 12.3 g/dL. (**a**) Progression-free survival (PFS). (**b**) Overall survival (OS).

**Figure 4:**
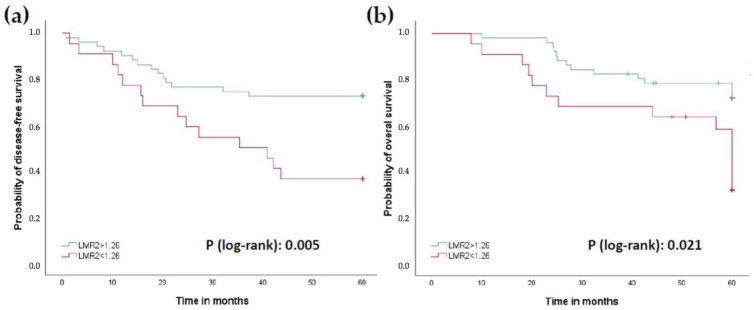
Prognostic role of LMR2 levels with a cut-off value of 1.26. (**a**) Progression-free survival (PFS). (**b**) Overall survival (OS).

**Figure 5:**
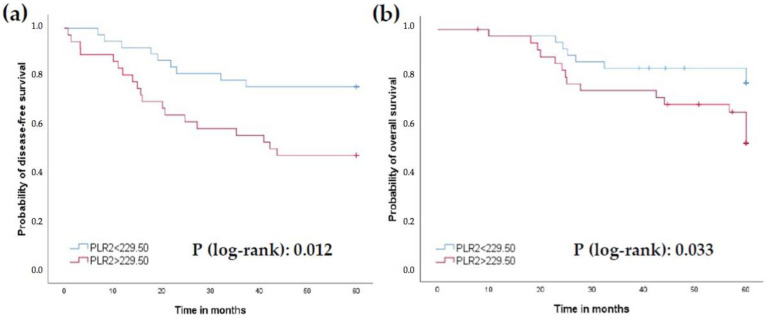
Prognostic role of PLR2 levels with a cut-off value of 229.50. (**a**) Progression-free survival (PFS). (**b**) Overall survival (OS).

Patients with Hb2 levels lower than 12.3 g/dL had worse 5-year survival PFS (35% vs. 71.7%) and OS (50% vs. 73.6%) compared to those with levels higher than 12.3 g/dL (*p* = 0.001 and 0.024, respectively; Figure 3a,b). Similar findings were obtained with LMR2 levels lower than 1.26 (5-year PFS 36.4% vs. 72.5% and OS 50% vs. 74.5%; *p* = 0.005 and 0.021, respectively; Figure 4a,b), and PLR2 levels greater than 229.50 (5-year PFS 47.2% vs. 75.7%; 5-year OS 55.6% vs. 78.4%; *p* = 0.012 and 0.033, respectively; Figure 5a,b).

In the multivariate analysis, we used logistic regression to evaluate which independent variables with statistically significant differences in the univariate analysis could be eliminated from the analysis (Table 4). We found that the presence of perineural invasion (*p* = 0.009; 95% CI 2.249–315.037), the presence of lymph node involvement (*p* = 0.007; 95% CI 2.555–397.000), Hb2 levels lower than 12.3 g/dL (*p* = 0.049; 95% CI 1.013–54.509), and LMR2 lower than 1.26 (*p* = 0.037; 95% CI 1.115–34.636) maintained their statistical significance.

**Figure 6:**
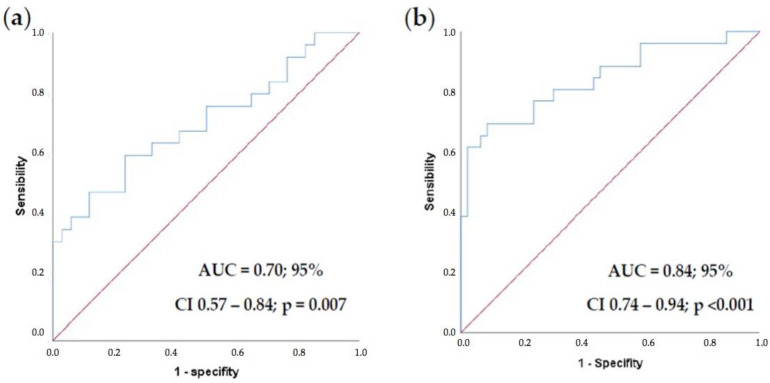
ROC curves for relapse (**a**) by Hb2, LMR2, and PLR2. (**b**) by Hb2, LMR2, PLR2, and N+.

We decided to create a model using the continuous variables obtained in the second blood draw (Hb2, LMR2, and PLR2). The model with these variables demonstrated acceptable ability to discriminate between groups (AUC = 0.707; 95% CI 0.57–0.84; *p* = 0.007) (Figure 6a). Given the known significance of lymph node involvement as a prognostic factor, we decided to add the number of metastatic lymph nodes (N+) to this model. The model demonstrated an excellent ability to discriminate (AUC = 0.84; 95% CI 0.74–0.94; *p* < 0.001) (Figure 6b).

Furthermore, the prognostic role of TRG in survival was analyzed. For this purpose, we stratified the patients into two groups based on their TRG (Good = TRG0 and TRG1; Poor = TRG2 and TRG3). No statistically significant differences were observed in PFS at 5 years (*p* = 0.496) or in OS at 5 years (*p* = 0.847) when comparing patients with good response versus poor response to CRT (Supplementary Figure S1a,b). In those patients who presented pathological complete response (TRG0), no significant differences were found either in 5-year PFS (*p* = 0.687) or OS (*p* = 0.961) compared to those who did not achieve a pathological complete response (nonTRG0) (Supplementary Figure S2a,b). 

### 3.3. Predictive Factors

We analyzed the clinical, pathological, and analytical parameters obtained before and after nCRT treatment of the groups stratified according to TRG (Supplementary Tables S2 and S3). Univariate analysis showed that only urea and bilirubin levels prior to the start of neoadjuvant treatment showed statistically significant differences between the two groups. ROC analysis showed poor capacity for the urea variable (AUC = 0.65; 95% CI 0.52–0.79; *p* = 0.035) but acceptable discrimination capacity for bilirubin (AUC = 0.75; 95% CI 0.62–0.88; *p* = 0.001) (Supplementary Figure S3a,b). With these results, a model was created that included both variables, but no improvement in discrimination capacity was obtained (AUC = 0.74; 95% CI 0.61–0.88; *p* = 0.001).

## 4. Discussion

In this study, we have found that low hemoglobin levels, a low lymphocyte/monocyte ratio, a high platelet/lymphocyte ratio, and a larger number of positive nodes after surgery associated with LARC relapse. In addition, KRAS mutations were associated with a poor prognosis at 5 years, and bilirubin levels were able to predict response to nCRT.

KRAS mutation status had been considered an important prognostic and predictive biomarker for colorectal cancer patients [19]. However, few studies have investigated the role of KRAS mutations in patients with LARC. Our results showed that KRAS mutations were present in 24.7% of patients, a lower frequency than reported in other publications [20,21,22]. In our study, we found that KRAS mutations were significantly associated with poorer OS and PFS in LARC patients receiving nCRT, but there was no relation between KRAS mutations and TRG. These findings are consistent with a recent meta-analysis [23].

The role of elevated hemoglobin levels before and during preoperative CRT treatment in rectal cancer has been associated in some studies with better local control [24], and lower mortality [25] for patients without anemia, considering a cut-off point at 12 g/dL. In our series, the relapse group persistently presented lower Hb levels than the control group, although they did not fall below the 12 g/dL cut-off until the post-surgery analysis (where the control group also fell below that level). Therefore, we believe that the cut-off for hemoglobin levels must be differently determined depending on the moment of sample analysis.

An elevated LMR has been identified as a prognostic factor for longer survival in colorectal cancer [26,27]. More specifically, in rectal cancer, a worse prognosis has been associated with a lower lymphocyte/monocyte ratio prior to neoadjuvant treatment [28]. On the other hand, an elevated PLR has been described as a poor prognostic factor in advanced colorectal cancer [29]. In rectal cancer, an elevated PLR, also determined before CRT, has been shown to be a poor prognostic factor [30], sometimes together with the NLR [31], although this could not be confirmed in other studies [32].

We need to point out that, in the previously referenced studies, the ratios were calculated at the baseline extraction prior to CRT (our blood draw 1). Although there are few studies investigating longitudinal changes in blood cell count ratios in CRC [33,34,35,36], there are no similar studies in LARC. In our case, the ratios that have been shown to play a prognostic role were obtained after CRT therapy and before surgery, that is, at the second blood draw (LMR2 and PLR2). Our study confirms the usefulness of these two parameters as prognostic biomarkers in LARC when they are analyzed at the right moment. Regarding the tumoral regression grading, many studies consider it a surrogate marker of survival, associating a better prognosis with a greater response obtained after neoadjuvant treatment [5,6,7,8,37,38,39]. However, we have not been able to corroborate this aspect in our study, as we did not find statistically significant differences either in 5-year PFS or OS between the groups stratified according to their TRG. One of the possible explanations is that this may be due to the regression grading system used (modified Ryan score [13] in our case) since in the aforementioned bibliography each study used a different grading system.

Regarding the search for potential predictive biomarkers of response to nCRT based on clinical-pathological and analytical characteristics, our study has only managed to establish differences in the levels of urea and bilirubin between both groups, with only bilirubin providing an acceptable sensitivity (AUC = 0.74), although it seems insufficient for clinical use. In the literature, elevated levels of bilirubin have been correlated with a higher risk of lymph node involvement after surgery [40,41], although the proposed cut-off level (2.6 mmol/L = 0.15 mg/dL) was widely exceeded by the two groups in our study and therefore does not seem to be comparable.

Regarding sample power, in the univariate analyses we found enough sample power for several of the comparisons. For example, in the case of hemoglobin values between the relapse (*n* = 32) and no relapse (*n* = 45) groups, power was 84% for differences equal or higher than 1.1. For PLR2, power was 86% for differences higher than 150. For calculating sample number in logistic regression models, the recommendation is to have ten events per covariable [42]. In our case, the relapse group had 32 variables, so the model could use a maximum of 3 covariables. In Table 4, we used KRAS status and 7 covariables. Although the odds ratios’ confidence intervals were ample, there was no problem in the convergence of the model.

Among the limitations of our study, it is necessary to emphasize the small number of patients and the fact that a single center was involved. In addition, the well-known tumor heterogeneity of colorectal cancer [20,43,44] may be the cause for a high variability.

## 5. Conclusions

The findings of this study allow us to confirm that KRAS mutations are associated with a poor prognosis in LARC. We propose a robust prognostic model to determine the risk of relapse in patients with LARC based on the number of positive lymph nodes after surgery, the hemoglobin levels, and the LMR and PLR ratios obtained after neoadjuvant therapy. In addition, bilirubin could have a modest predictive role in response to preoperative treatment. However, both hypotheses need to be confirmed in randomized studies with a larger number of patients.

## Figures and Tables

**Table 1:** Univariate analysis comparing groups.

Factor	Relapse Group	No Relapse Group	*p*-Value
Number of patients, *n* (%)	31 (40)	46 (60)	
Age at diagnosis (median/sd)	63.5/9.5	61.7/10.5	0.445
Male:Female (*n*)	23:8	34:12	1.000
cTNM ≥ T3N0, *n* (%)	29 (40)	44 (60)	1.000
MSI, *n* (%)	2 (22)	7 (78)	0.301
KRAS mutations, *n* (%)	12 (63)	7 (37)	**0.028**
Low LARC (0–5 cm), *n* (%)	11 (34)	22 (66)	0.350
Medium LARC (6–10 cm), *n* (%)	15 (43)	20 (57)	0.816
Upper LARC (11–15 cm), *n* (%)	5 (56)	4 (44)	0.472
Smoking history, *n* (%)	16 (39)	25 (61)	0.821
CEA levels, ng/mL (median/sd)	14.9/18.3	7.4/13.6	0.057
Neoadjuvant 5-FU/LVci, *n* (%)	20 (38)	32 (62)	0.619
TDSNT, days (mean/sd)	53.1/18.7	54.3/15.9	0.762
TENTS, days (mean/sd)	48.4/9.2	47.6/11.3	0.732
TDS, days (mean/sd)	140.8/22.6	141.2/21.4	0.943
TSAC, days (mean/sd)	59.7/38.0	46.33/17.1	0.112
APA, *n* (%)	16 (48)	17 (52)	0.236
ypTNM ≥ T3N0, *n* (%)	20 (56)	16 (44)	**0.019**
TRG0, *n* (%)	2 (29)	5 (71)	0.703
TRG1, *n* (%)	7 (33)	14 (67)	0.791
TRG2, *n* (%)	11 (33)	22 (67)	0.630
TRG3, *n* (%)	8 (62)	5 (38)	0.065
EMVI, *n* (%)	6 (75)	2 (25)	**0.048**
Lymphatic invasion, *n* (%)	7 (78)	2 (12)	**0.023**
Perineural invasion, *n* (%)	13 (87)	2 (13)	**<0.001**
Lymph node involved, *n* (%)	14 (70)	6 (30)	**0.001**
Positive lymph nodes (mean/sd)	3/5.8	0.3/1.2	**0.001**
Resected lymph nodes (mean/sd)	10.0/7.0	8.8/5.5	0.600

Abbreviations: MSI: Microsatellite Instability. TDSNT: Time from diagnosis to start of neoadjuvant treatment. TENTS: Time from the end of neoadjuvant treatment to surgery. TDS: Time from diagnosis to surgery. TSAC: Time from surgery to start of aCRT. APA: Abdomino-perineal amputation. EMVI: Extramural venous invasion. Statistically significant differences are indicated in bold.

**Table 2:** Univariate analysis of hematologic parameters.

Characteristics	Relapse Group	No Relapse Group	*p*-Value
Leukocyte 1, c/mm^3^ (mean/sd)	8146.0/3413.2	7697.5/1914.1	0.930
Leukocyte 2, c/mm^3^ (mean/sd)	5927.1/3491.2	5195.5/1277.0	0.781
Leukocyte 3, c/mm^3^ (mean/sd)	6448.1/2129.8	6733.3/2567.8	0.754
Leukocyte 4, c/mm^3^ (mean/sd)	5305/1599.1	5488.1/1755.5	0.694
Neutrophil 1, c/mm^3^ (mean/sd)	5124.6/2872.8	4500/1390.6	0.493
Neutrophil 2, c/mm^3^ (mean/sd)	4262.5/3173.8	3364.4/1085.0	0.812
Neutrophil 3, c/mm^3^ (mean/sd)	4576.6/1887.2	4912.1/2500.6	0.839
Neutrophil 4, c/mm^3^ (mean/sd)	3555/1396.4	3590.4/1535.2	0.898
Lymphocyte 1, c/mm^3^ (mean/sd)	2140/798.2	2300/850.6	0.437
Lymphocyte 2, c/mm^3^ (mean/sd)	859.2/382.0	1051.1/486.4	0.076
Lymphocyte 3, c/mm^3^ (mean/sd)	1007.0/529.0	1004.0/506.0	0.981
Lymphocyte 4, c/mm^3^ (mean/sd)	1070/432.9	1178.5/481.1	0.395
Monocyte 1, c/mm^3^ (mean/sd)	654.6/256.9	630/224.4	0.746
Monocyte 2, c/mm^3^ (mean/sd)	599.6/236.0	520/173.9	0.267
Monocyte 3, c/mm^3^ (mean/sd)	592.2/298.8	579.0/188.9	0.433
Monocyte 4, c/mm^3^ (mean/sd)	490/137.2	535.7/193.5	0.451
Hemoglobin 1, g/dL (mean/sd)	13.3/1.9	14.1/1.6	0.055
Hemoglobin 2, g/dL (mean/sd)	12.5/1.7	13.5/1.3	**0.008**
Hemoglobin 3, g/dL (mean/sd)	10.9/1.5	11.9/1.5	**0.007**
Hemoglobin 4, g/dL (mean/sd)	13.4/1.7	13.4/1.3	0.792
Platelets 1, c/mm^3^ (mean/sd)	267,285.7/114,369.7	238,700/53,993.4	0.231
Platelets 2, c/mm^3^ (mean/sd)	264,428.5/133,926.5	206,844.4/40,279.6	0.200
Platelets 3, c/mm^3^ (mean/sd)	326,851.8/144,934.3	272,285.7/97,952.2	0.176
Platelets 4, c/mm^3^ (mean/sd)	205,100/78,874.9	201,119.0/41,163.0	0.834

Statistically significant differences are indicated in bold.

**Table 3:** Univariate analysis of hematologic ratios.

Characteristics	Relapse Group	No Relapse Group	*p*-Value
Neutrophil-Lymphocyte Ratio 1 (mean/sd)	2.5/1.2	2.2/1.3	0.118
Neutrophil-Lymphocyte Ratio 2 (mean/sd)	5.8/4.7	3.8/2.2	0.075
Neutrophil-Lymphocyte Ratio 3 (mean/sd)	5.9/4.1	6.4/5.4	0.912
Neutrophil-Lymphocyte Ratio 4 (mean/sd)	3.8/2.0	3.5/2.2	0.503
Lymphocyte-Monocyte Ratio 1 (mean/sd)	3.5/1.6	3.8/1.4	0.448
Lymphocyte-Monocyte Ratio 2 (mean/sd)	1.5/0.6	2.2/1.5	**0.011**
Lymphocyte-Monocyte Ratio 3 (mean/sd)	1.8/0.8	1.8/0.8	0.956
Lymphocyte-Monocyte Ratio 4 (mean/sd)	2.2/0.8	2.4/1.3	0.786
Platelet-Lymphocyte Ratio 1 (mean/sd)	137.8/68.4	117.9/54.2	0.206
Platelet-Lymphocyte Ratio 2 (mean/sd)	386.2/290.9	236.4/114.7	**0.029**
Platelet-Lymphocyte Ratio 3 (mean/sd)	386.3/213.6	331.5/187.8	0.238
Platelet-Lymphocyte Ratio 4 (mean/sd)	217.5/103.1	200.9/89.7	0.519

Statistically significant differences are indicated in bold.

**Table 4:** Logistic regression based on relapse.

Predictor	Β	SE β	Χ^2^	*p*	OR	95%CI	
						Lower	Upper
KRAS status	0.803	1.047	0.588	0.443	2.232	0.287	17.381
EMVI	1.382	2.269	0.371	0.543	3.983	0.047	340.268
LVI	−0.946	2.578	0.135	0.714	0.388	0.002	60.708
PNI	3.282	1.261	6.775	**0.009**	26.619	2.249	315.037
ypN	3.461	1.287	7.230	**0.007**	31.851	2.555	397.000
Hb2 < 12.3	2.006	1.017	3.892	**0.049**	7.432	1.013	54.509
LMR2 < 1.26	1.827	0.877	4.343	**0.037**	6.214	1.115	34.636
PLR2 > 229.50	1.867	1.006	3.448	**0.063**	6.469	0.901	46.425

Abbreviations: EMVI: Extramural venous invasion. LVI: Lymphovascular invasion. PNI: Perineural invasion. ypN: pathological lymph node stage after treatment. Statistically significant differences are indicated in bold.

## Data Availability

The data presented in this study are available on request from the corresponding author.

## Supplementary Materials

The following supporting information can be downloaded at: https://www.mdpi.com/article/10.3390/jcm11206091/s1, Figure S1: Impact of TRG on progression free survival (S1a) and overall survival (S1b). Blue: Tumoral Response Grade 0–1. Red: Tumoral response grade 2–3. Figure S2: Impact of Tumoral Grade Response 0 on progression free survival (S2a) and overall survival (S2b). Figure S3: ROC analyses for predictive factors in relation to Tumoral Response Grade. S3a: by urea. S3b: by bilirubin. Table S1: Univariate analysis of biochemical parameters. Table S2: Patient demographics based on tumoral response grading. Table S3: Hematologic parameters based on tumoral response grading.
