# Supplementary material for: Prognostic and Predictive Biomarkers in Patients with Locally Advanced Rectal Cancer (LARC) Treated with Preoperative Chemoradiotherapy
Source: J Clin Med. 2022 Oct 16;11(20):6091. doi: 10.3390/jcm11206091 (PMC9604791; doi:10.3390/jcm11206091)
Supplement: Supplementary file 1 [file jcm-11-06091-s001.zip › Figure S1-S2.pptx]

## Slide 1
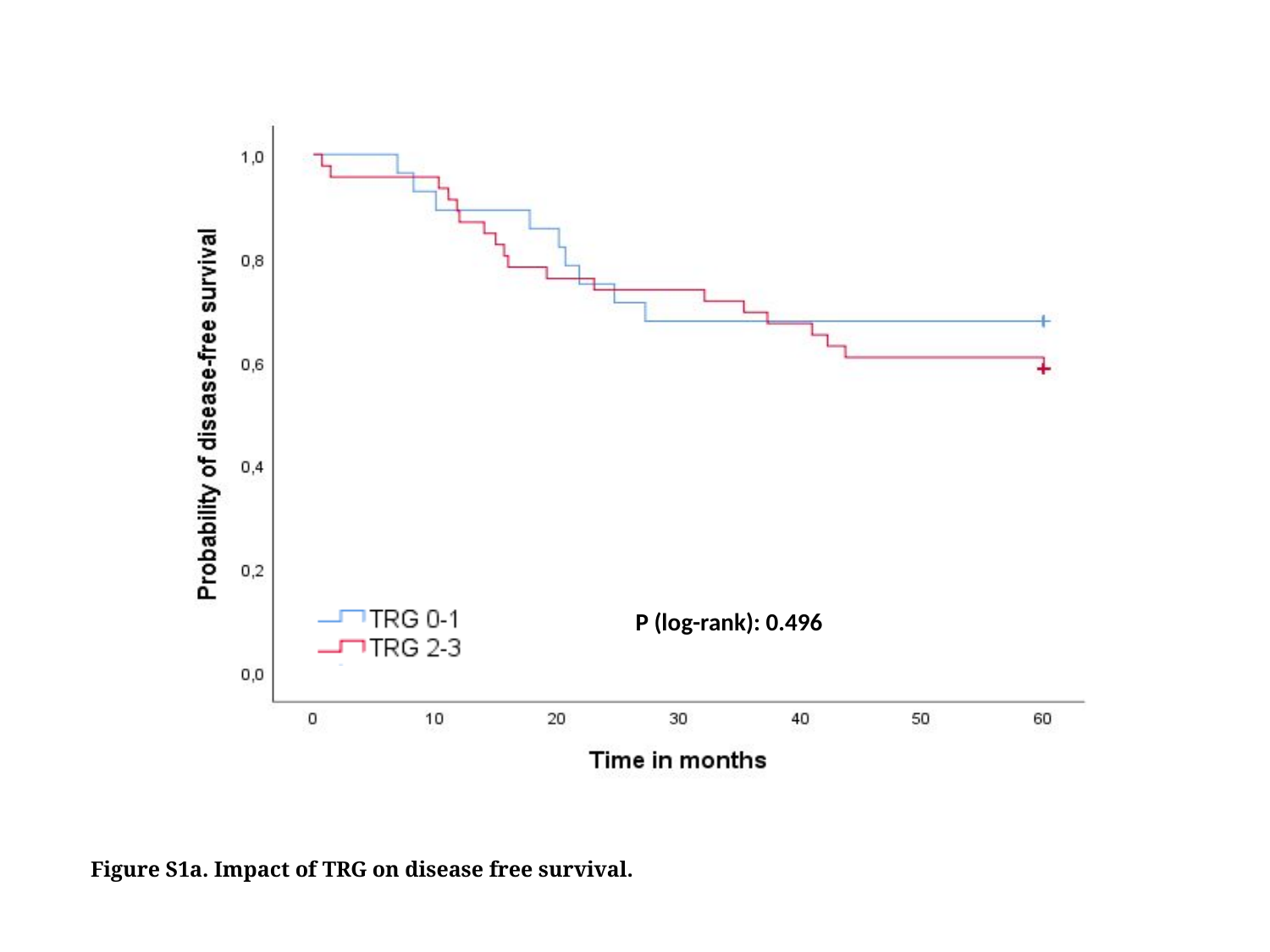

P (log-rank): 0.496
Figure S1a. Impact of TRG on disease free survival.

## Slide 2
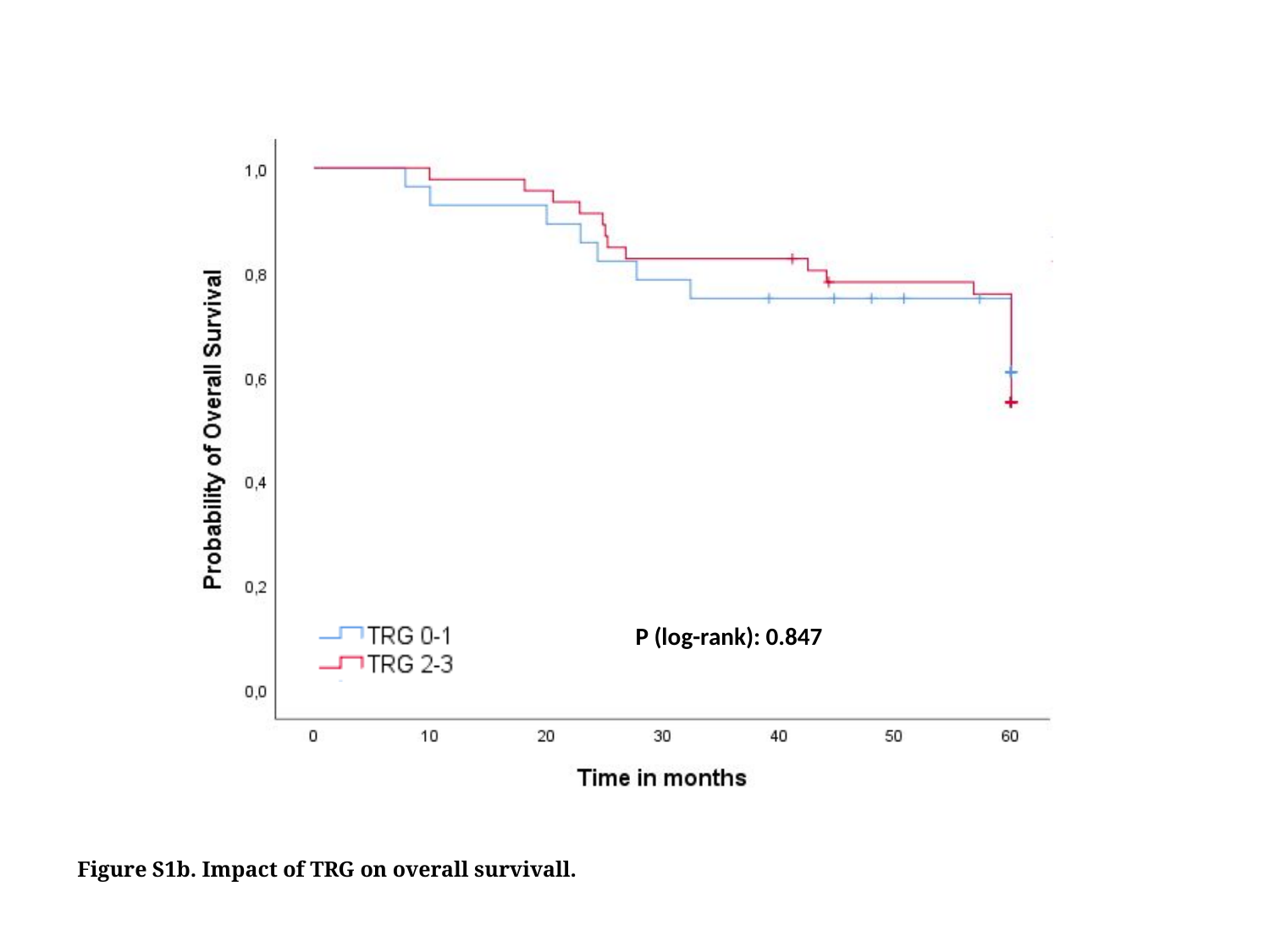

P (log-rank): 0.847
Figure S1b. Impact of TRG on overall survivall.

## Slide 3
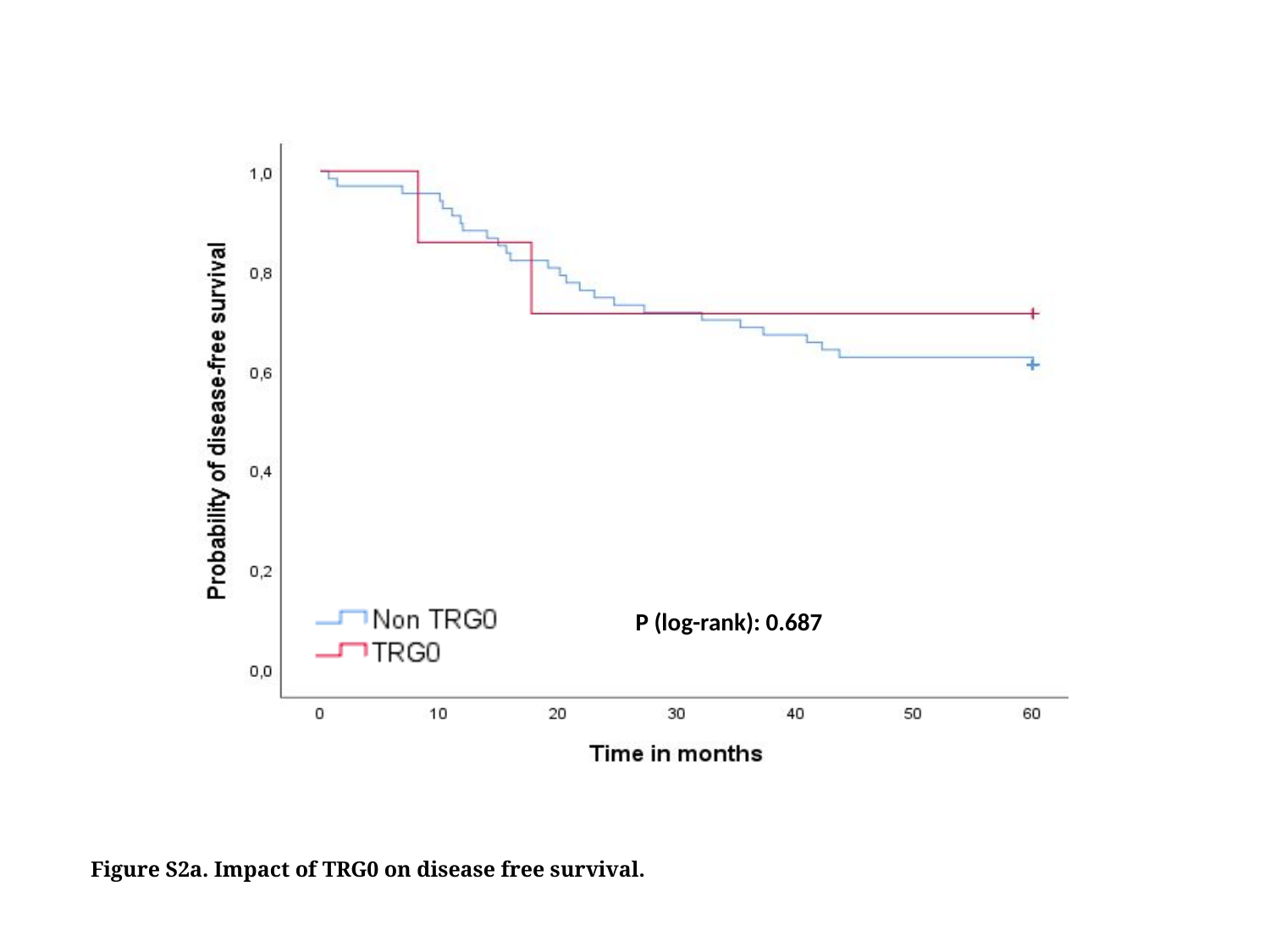

P (log-rank): 0.687
Figure S2a. Impact of TRG0 on disease free survival.

## Slide 4
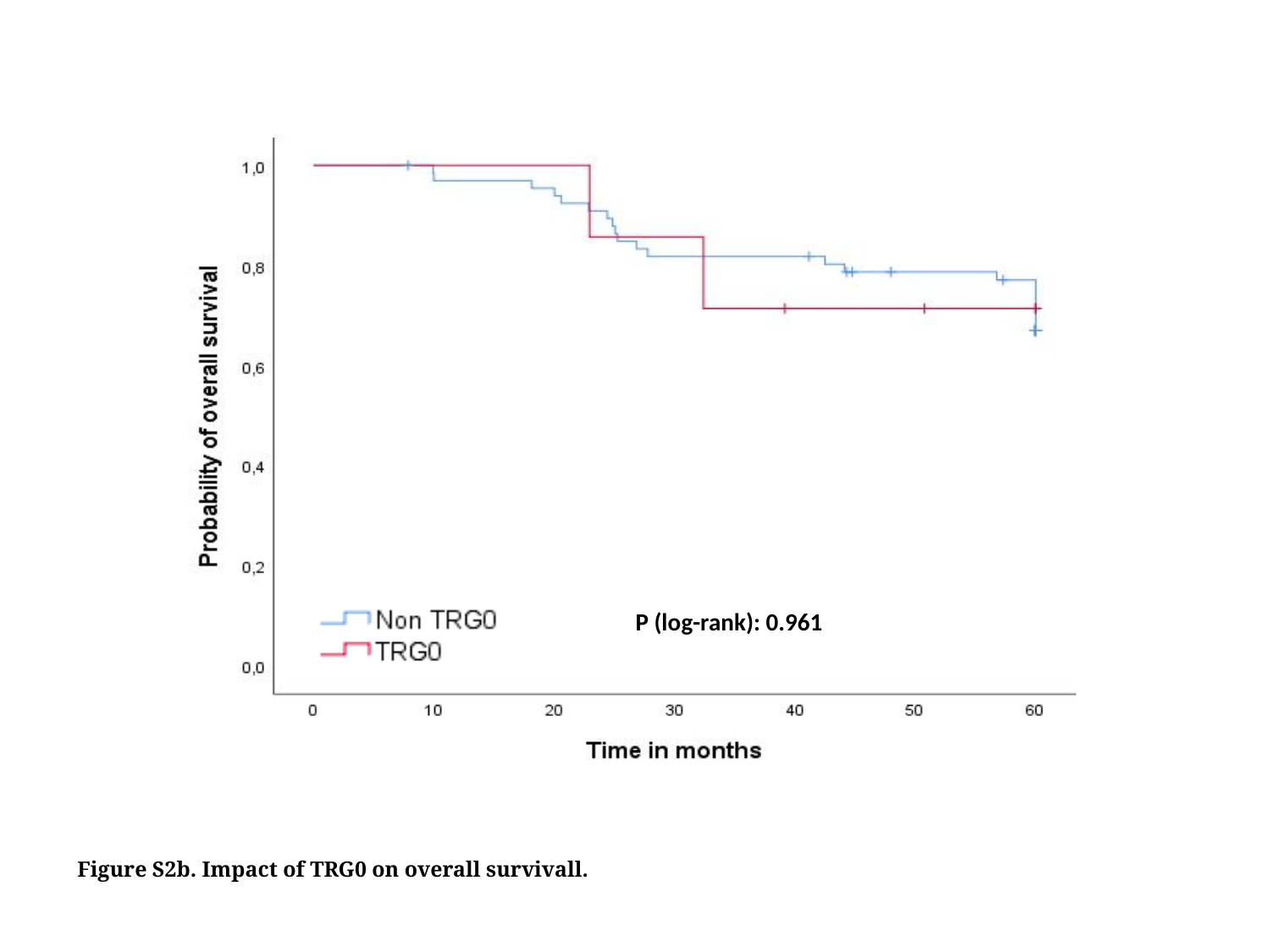

P (log-rank): 0.961
Figure S2b. Impact of TRG0 on overall survivall.
