# Supplementary material for: Prognostic and Predictive Biomarkers in Patients with Locally Advanced Rectal Cancer (LARC) Treated with Preoperative Chemoradiotherapy
Source: J Clin Med. 2022 Oct 16;11(20):6091. doi: 10.3390/jcm11206091 (PMC9604791; doi:10.3390/jcm11206091)
Supplement: Supplementary file 1 [file jcm-11-06091-s001.zip › Figure S3.pptx]

## Slide 1
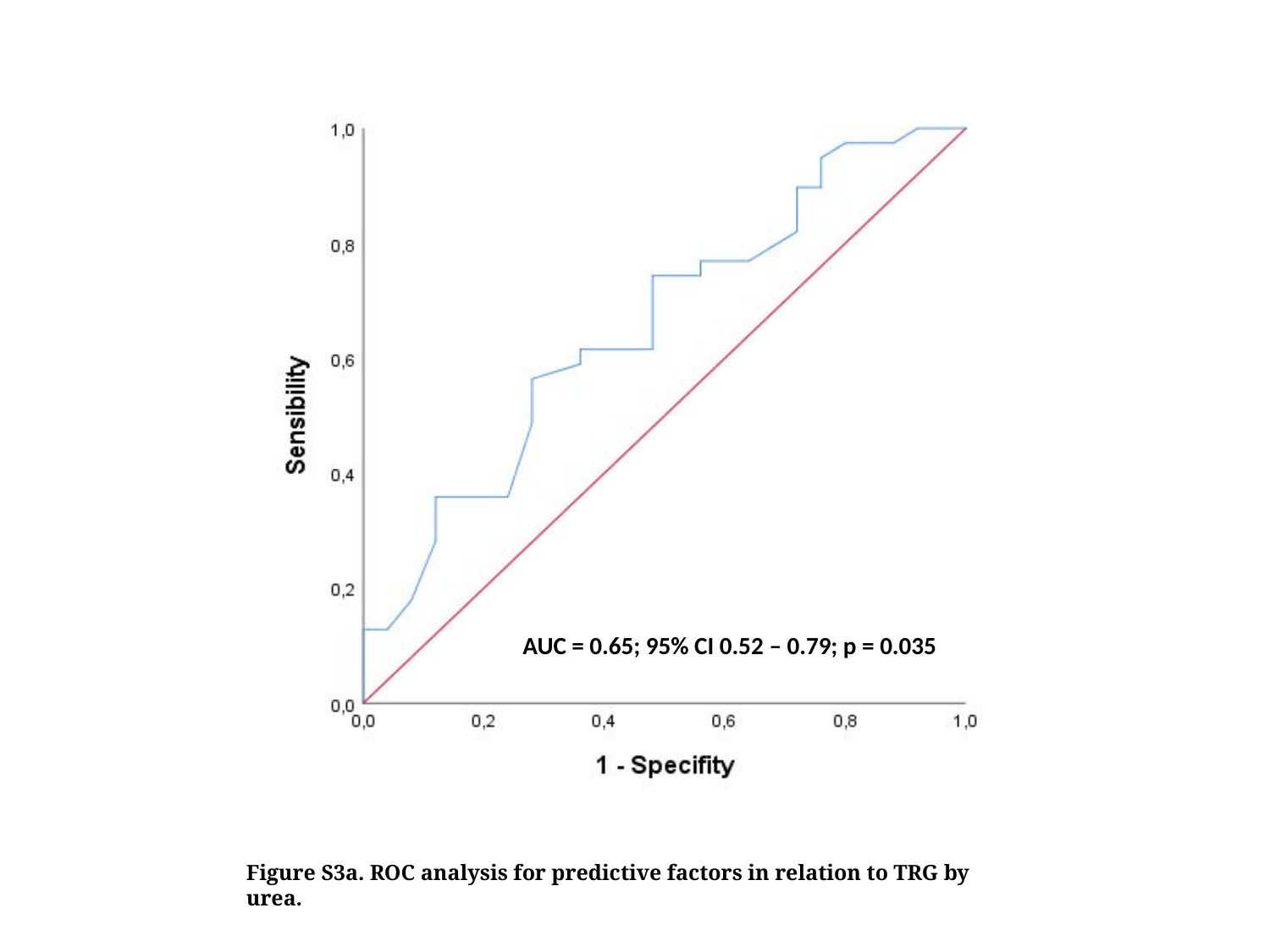

AUC = 0.65; 95% CI 0.52 – 0.79; p = 0.035
Figure S3a. ROC analysis for predictive factors in relation to TRG by urea.

## Slide 2
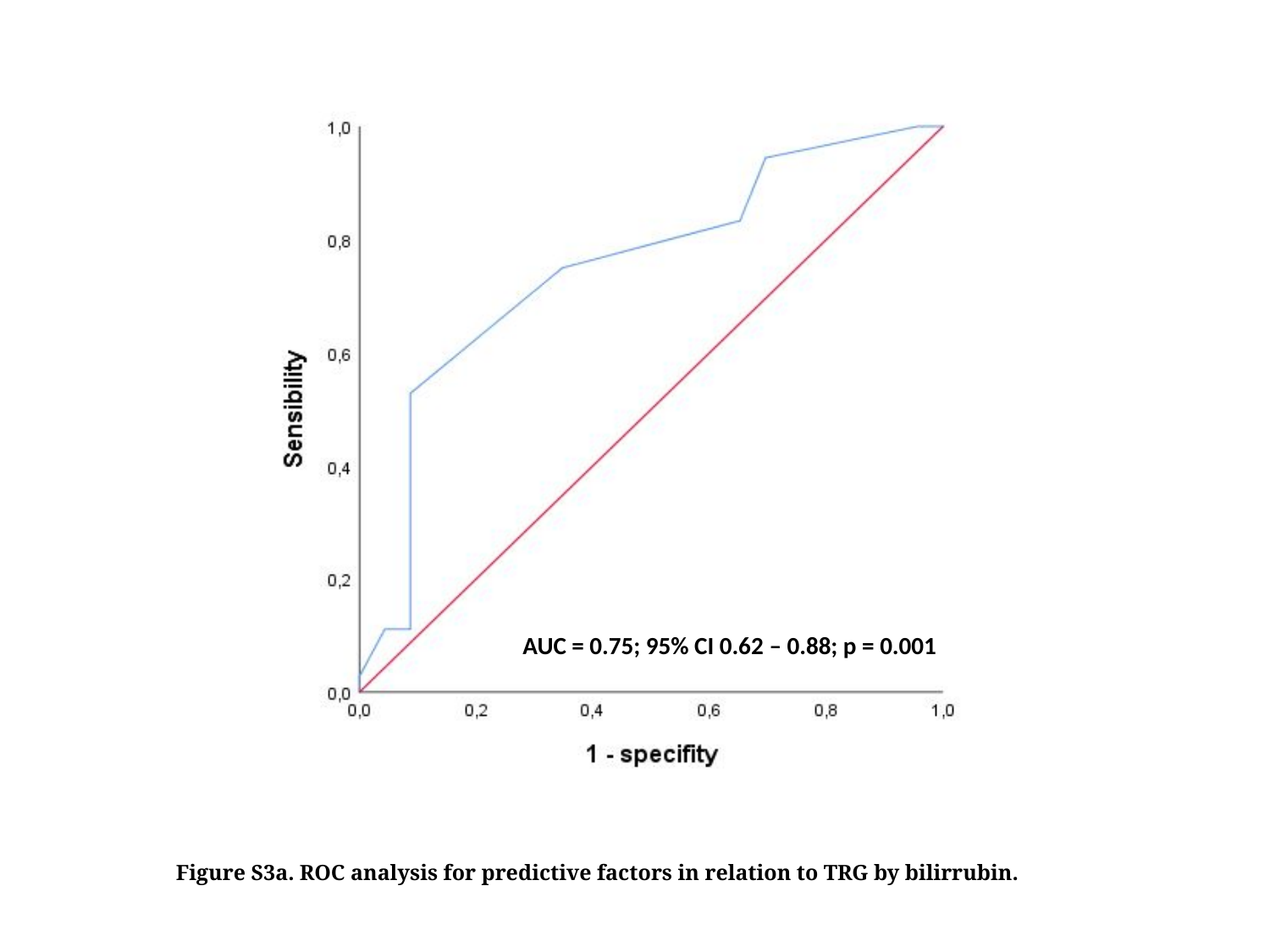

AUC = 0.75; 95% CI 0.62 – 0.88; p = 0.001
Figure S3a. ROC analysis for predictive factors in relation to TRG by bilirrubin.
